# Mortality rate and predictors of colorectal cancer patients in Ethiopia: a systematic review and meta-analysis

**DOI:** 10.1186/s12885-024-12597-9

**Published:** 2024-07-10

**Authors:** Zewdu Bishaw Aynalem, Abebaw Bires Adal, Temesgien Fentahun Ayele, Gashaw Melkie Bayeh, Almaw Genet Yeshiwas, Tadesse Miretie Dessie, Tilahun Degu Tsega

**Affiliations:** 1 Department of Nursing, College of Medicine and Health Sciences, Injibara University, Injibara, Ethiopia; 2 Department of Environmental Health, College of Medicine and Health Sciences, Injibara University, Injibara, Ethiopia; 3 Department of Public Health, College of Medicine and Health Sciences, Injibara University, Injibara, Ethiopia

**Keywords:** Colorectal cancer, Mortality, Predictors, Ethiopia

## Abstract

**Introduction:**

The incidence of colorectal cancer (CRC) has been increasing in Sub-Saharan countries, including Ethiopia. However, the real mortality rate for CRC patients in Ethiopia has not been established. Therefore, this systematic review and meta-analysis aimed to determine the overall mortality rate and identify predictors among CRC patients in Ethiopia.

**Methods:**

PubMed, EMBASE, Web of Science, Scopus, Science Direct, and Google Scholar were searched to identify relevant articles. The preferred reporting items for systematic reviews and meta-analyses (PRISMA) were followed. The quality of the included studies was assessed using the Newcastle-Ottawa Scale Critical Appraisal checklist. A random effect model was used to estimate the pooled mortality rate and adjusted hazard ratio (AHR). Publication bias was assessed using funnel plots and Egger’s regression test, while heterogeneity was evaluated through the Cochran Q test and I^2^ statistics.

**Results:**

After reviewing 74 articles, only 7 studies met the criteria and were included in the analysis. The analysis revealed that the overall mortality rate among CRC patients in Ethiopia was 40.5% (95% confidence interval [CI]: 32.05, 48.87) while the survival rates at 1 year, 3 years, and 5 years were 82.3% (95% CI: 73.33, 91.31), 48.8% (95% CI: 43.35, 54.32), and 26.6% (95% CI: 21.26, 31.91) respectively. Subgroup analysis indicated that studies conducted after 2017 had higher mortality rates compared to those studied earlier (43.0% vs. 38.2%). Older age (AHR: 1.89, 95% CI: 1.27, 2.82); being married (AHR: 2.53, 95% CI: 1.79, 3.57); having comorbidities (AHR: 1.84, 95% CI: 1.45, 2.35); having high CEA levels (AHR: 2.06, CI: 1.35, 3.13); being in stage II (AHR: 4.13, 95% CI: 1.85, 9.22), III (AHR: 8.62, 95% CI: 3.88, 19.15), and IV (AHR: 8.06, CI: 2.89, 22.49) were the most important predictors.

**Conclusion:**

In Ethiopia, the mortality rate among individuals diagnosed with CRC is high, with two out of five patients dying from this disease. Age, marital status, CEA level, comorbidities, and cancer stage were identified as predictors of mortality in CRC patients. Therefore, early detection and screening should be prioritized, particularly for older patients, those who are married, have comorbidities, elevated CEA levels, and advanced cancer stages.

**Supplementary Information:**

The online version contains supplementary material available at 10.1186/s12885-024-12597-9.

## Introduction

Colorectal cancer (CRC), an umbrella term encompassing colon, rectal, and anal cancer, is a major public health problem worldwide [1, 2]. It’s more common in developed countries, but it’s on the rise in middle and low-income countries because of westernization [3]. In recent years, the number of cases has been growing at an alarming rate, with about 1.93 million new cases and 935,000 deaths globally [4, 5]. Among these deaths, 46.3% were recorded in Asia, while Europe, North America, and Africa recorded 29.7%, 8.4%, and 4.6% respectively [6]. These numbers collectively make up about 9% of all cancer-related deaths [7].

It is estimated that by 2030, the worldwide burden of CRC will upsurge by 60%, reaching more than 2.2 million new cases and 1.1 million deaths [8]. In the same vein, by the year 2040, there will be 1.6 million deaths from colorectal cancer, an increase of 63% [9]. What’s concerning is that this surge is primarily affecting young adults under 50, who play a crucial role in economic growth and societal development [10]. Despite declining rates among older adults in the past 10 years [11], CRC is now the leading cause of cancer-related deaths in men and the second leading cause in women. In the late 1990s, it was the fourth leading cause of cancer-related deaths in individuals under 50 [12]. The number of young adults dying from this cancer has been increasing by 1.3% every year, emphasizing the need for urgent action to combat this disease [13].

Colorectal cancer mortality rates differ from country to country due to variations in healthcare systems, early detection programs, lifestyles, and socioeconomic status [14, 15]. For example, in the United States, over 50,630 patients lost their lives due to CRC in 2018, ranking it second next to lung cancer [16]. In China, the number of deaths from CRC in 2020 was approximately 250,000, making up about 28.11% of all deaths [2]. Malaysia recorded that among 4,501 cases, 44.7% of deaths were attributed to colorectal cancer [17]. Sub-Saharan Africa has the highest proportion of colorectal cancer-related deaths globally [18]. This is primarily because the majority of colorectal cancers in this region are detected at advanced and late stages with limited treatment options [19–21]. Additionally, multiple predictors, such as patient demographics, cancer stage, histological characteristics, comorbidities, treatment modalities, lifestyles, and socioeconomic factors might be attributed to this high mortality [8, 22–24].

In recent times, there has been significant advancement in understanding the pathogenesis, screening, diagnosis, and treatment of CRC. Despite these improvements, CRC mortality remains high in low- and middle-income countries, including Ethiopia [25–28]. In Ethiopia, although some primary studies have reported CRC mortality rate and predictors, there is a lack of comprehensive research on the national mortality rate and contributing factors. As a result, the true mortality rates for CRC in Ethiopia remain largely unknown.

Mortality figures for colorectal cancer are primarily estimated from population-based cancer registries and vital registration sources. The establishment of such a system is crucial for acquiring accurate data on cancer trends, treatment effectiveness, and overall outcomes. Establishing and using such systems is essential for acquiring accurate data on cancer trends, treatment effectiveness, and overall health outcomes and serves as the backbone for informed decision-making in healthcare and research. Unfortunately, Ethiopia at present has only one population-based cancer registry, which covers a mere 3–5% of the total population. This registry is only confined to small urban areas, lacking comprehensive coverage of cancer statistics from rural areas or the entire nation. As well as the quality of mortality data is poor. Given the infancy stage of population-based cancer registration in Africa, including Ethiopia, individual cohort studies play a crucial role. They provide valuable insights into the cancer burden until a comprehensive national and regional cancer registry system is fully developed [29–32].

Determining the mortality rate and identifying the factors contributing to increased mortality rate among colorectal cancer patients is crucial for improving patient outcomes, personalizing treatment approaches, and guiding healthcare decision-making [33, 34]. Without concrete data, it is challenging for the government to allocate resources appropriately for CRC management compared to the support given to breast and cervical cancer management. As a result, this systematic review and meta-analysis aimed to fill the above mentioned gaps.

## Methods

### Reporting

The Preferred Reporting Items of Systematic Reviews and Meta-Analysis (PRISMA) checklist [35] was followed to conduct this review **(Supplementary Table 1).** In addition, the protocol was registered in PROSPERO with registration number CRD42024498810.

### Search strategy

PubMed, EMBASE, Web of Science, Scopus, Science Direct, and Google Scholar databases were searched to identify relevant articles from December 21–30, 2023. The search terms used were “colorectal cancer,” “colorectal neoplasm,” “colorectal tumor,” “colonic neoplasm,” “colon cancer,” “rectal cancer,” “outcomes of colorectal cancer,” “mortality,” “death,” “mortality rate,” “predictors,” “determinant,” “risk factors,” and “Ethiopia.” These search terms were combined using the Boolean operators “OR” and “AND.” The search was carried out from inception until December 30, 2023. To improve the search coverage, cross-references from the bibliographies of selected studies were also searched. In addition, articles with incomplete data were accessed by contacting the corresponding author. EndNote X8 was used to import all searched records and duplicates were then eliminated.

### Inclusion and exclusion criteria

This systematic review and meta-analysis encompassed the following studies: (1) primary studies that provided clear reports on either the mortality rates or predictors of colorectal cancer in Ethiopia; (2) all observational studies available electronically until December 30, 2023; (3) studies published in English language. However, studies that were expert opinions, case reports, conference presentations, articles without full access, studies that did not report the outcome of interest, and studies with methodological problems were excluded.

### Data extraction

Five authors (ZBA, TMD, GMB, AGY, and TDT) independently extracted all necessary data using this format. The data extraction format included author, year of publication, sample size, follow-up year, study quality, overall mortality rate, and the selected predictors of mortality from colorectal cancer. Any disagreement while data extractions were resolved through discussion.

### Quality assessment

To assess the quality of included studies, the Newcastle-Ottawa Scale (NOS) score for cohort studies was utilized as it is currently the most commonly used tool [36]. This tool consists of 3 distinct sections: selection (containing 4 items), comparison (containing 1 item), and outcome (containing 3 items). Studies were scored based on their overall scores and categorized into 3 groups: good quality (3 or 4 stars in the selection domain AND 1 or 2 stars in the comparability domain AND 2 or 3 stars in the outcome domain); fair quality (2 stars in the selection domain AND 1 or 2 stars in the comparability domain AND 2 or 3 stars in the outcome domain); and poor quality (0 or 1 star in the selection domain OR 0 star in the comparability domain OR 0 or 1 star in the outcome domain) **(Supplementary Table 2).** Only studies that scored five or more on the NOS were included in the analysis.

#### Outcome measurement

The outcome variable of the study was CRC mortality rate, which refers to the number of deaths attributed to CRC within a specific timeframe. The pooled mortality rate was calculated by dividing the total number of deaths that occurred during the follow-up period by the total number of observed patients, and then multiplying by 100.

#### Heterogeneity test and publication bias

Cochran Q-test and Higgins’s I^2^ test statistics were computed to assess heterogeneity among all studies. Accordingly, I^2^ results of 25%, 50%, and 75% represent low, medium, and high heterogeneity, respectively [37]. A random-effect model of analysis was used due to the high heterogeneity between studies [38]. A funnel plot and Egger’s test were used to assess publication bias at a significance level lower than 0.05 [39, 40]. The visual inspection of the funnel plot was approximately asymmetrical, suggesting publication bias **(Supplementary Fig. 1)**. Therefore, the Egger test was used as a statistical method to assess publication bias and found *p* = 0.097 (statistically not significant) indicating that there was no evidence of publication bias.

### Statistical analysis

The data was extracted using a Microsoft Excel spreadsheet and imported into STATA version 11 for analysis. A random effect meta-analysis model was used to estimate the pooled mortality rate. Statistical heterogeneity was evaluated by Cochran’s Q test and Higgins’s I^2^ statistics, with I^2^ values of 25%, 50%, and 75% indicating low, medium, and high heterogeneity, respectively. Subgroup analysis was performed based on factors like study year, sample size, and year of publication. Funnel plots were constructed to visually assess publication bias, with a p-value of < 0.05 indicating significant bias. Furthermore, Egger’s regression test was used to detect evidence of publication bias. A forest plot was used to present the pooled mortality rate of colorectal cancer. To identify predictors of colorectal cancer mortality, a hazard ratio for each factor was calculated. Finally, a p-value of less than 0.05 at 95% CI was used to declare statistical prediction.

## Results

### Selection and characteristics of the included studies

**Fig. 1:**
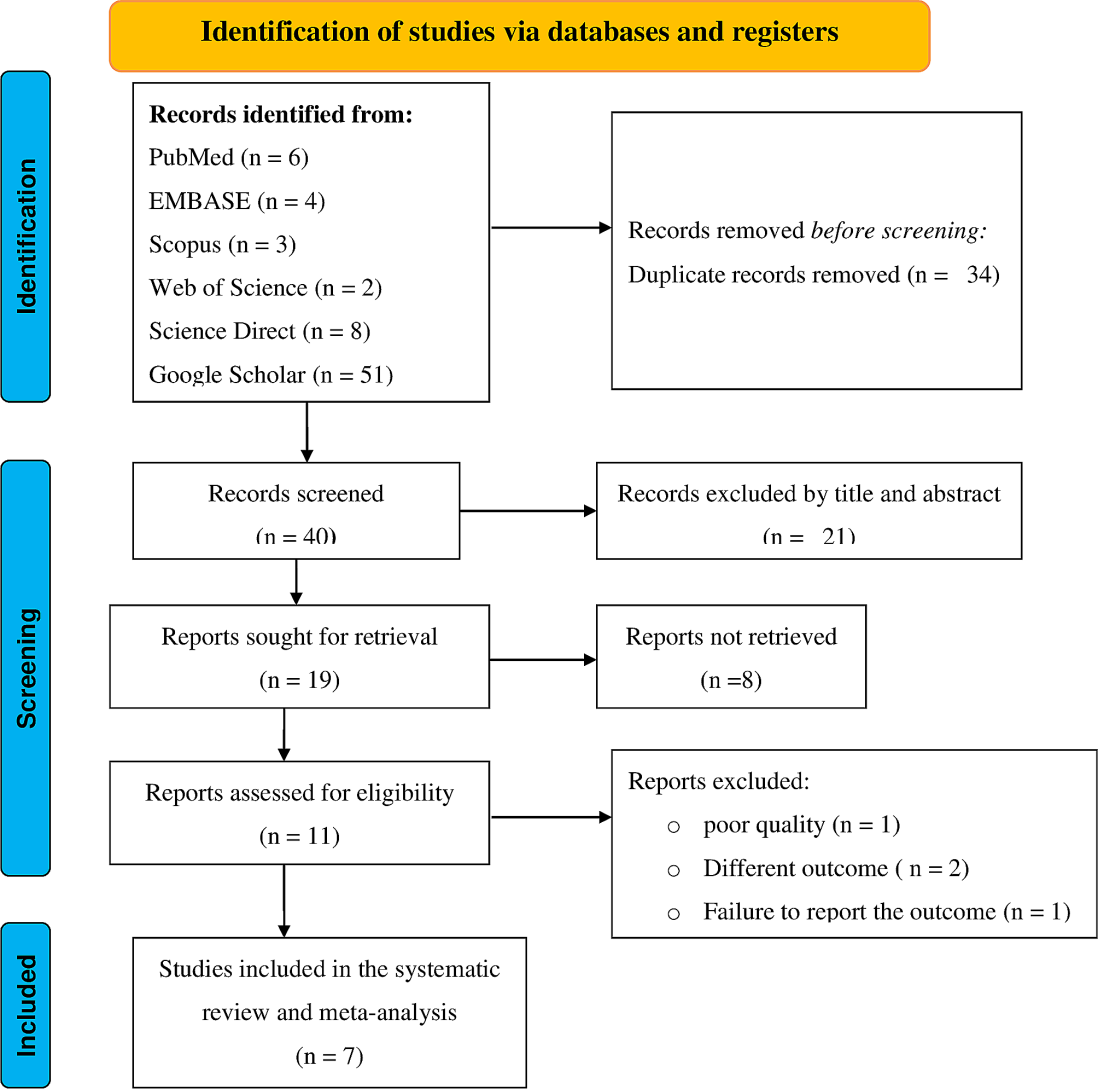
PRISMA flowchart diagram of the study selection

Initially, a total of 74 articles were retrieved from the PubMed, EMBASE, Web of Science, Scopus, Science Direct, and Google Scholar databases. 34 duplicates were found and removed. Of the remaining 40 articles, 21 were removed after careful examination of their titles and abstracts. Subsequently, 11 full-text articles were assessed, out of which 4 were excluded due to reasons such as poor quality, different outcomes, or failure to report the outcome of interest. Finally, a total of 7 studies that fulfilled the inclusion criteria were included in this systematic review and meta-analysis. The detailed retrieval process is shown in Fig. 1.

The general characteristics of the included studies are summarized in Table 1. These studies were conducted between 2013 and 2022 and involved a total of 2,539 patients diagnosed with colorectal cancer. Six of the included studies were retrospective cohorts [32, 41–45] and one was a prospective cohort study [46]. The sample sizes ranged from 161 [32] to 621 [41] participants. Geographically, six studies were conducted in Addis Ababa [32, 41–44, 46], while one study was conducted in the Amhara region [45]. In the included studies, the mean age of the participants was 46.27 (SD ± 17.13) years. More than half of them (55.57%) were male, while 49.52% had colon cancer and 41.21% had stage IV cancer. About 28.05% of the participants had a history of alcohol consumption, 16.57% were smokers, and 9.97% had a family history of colorectal cancer. Nearly one-fourth (22.95%) of them had preexisting comorbidities, of which 7.17% had high blood pressure, 4.13% had diabetes mellitus and 2.5% had HIV/AIDS. The overall mortality rate among colorectal patients ranged from 27.75 [45] to 67.46 [46].

**Table 1:** Characteristics of included studies

No	Author	*P*. Year	Study area	Study design	Sample size	Event	Mortality rate	Median survival	Survival rate (%)			Study Quality
									1 year	3 year	5 year	
1.	Atinafu et al. [41]	2020	Addis Ababa	Cohort	621	202	32.5	34.8	90.7	47	21.7	Good
2.	Atinafu et al. [42]	2022	Addis Ababa	Cohort	434	151	34.8	34.8	93.3	51.8	23.94	Good
3.	Etissa et al. [43]	2021	Addis Ababa	Cohort	422	175	41.5	39	80	55	33	Good
4.	Getabile et al. [44]	2022	Addis Ababa	Cohort	325	111	34.15	23	NR	NR	NR	Good
5.	Teka et al. [32]	2021	Addis Ababa	Cohort	161	75	46.6	21	NR	39.5	28.7	Good
6.	Tiruneh et al. [45]	2022	Amhara	Cohort	367	101	27.75	30.66	NR	NR	NR	Good
7.	Zingeta et al. [46]	2023	Addis Ababa	Cohort	209	141	67.46	17	63.16	NR	NR	Good

**Notes**: NR = not reported, P. Year = publication year

### Pooled mortality and survival rate among colorectal cancer patients in Ethiopia

**Fig. 2:**
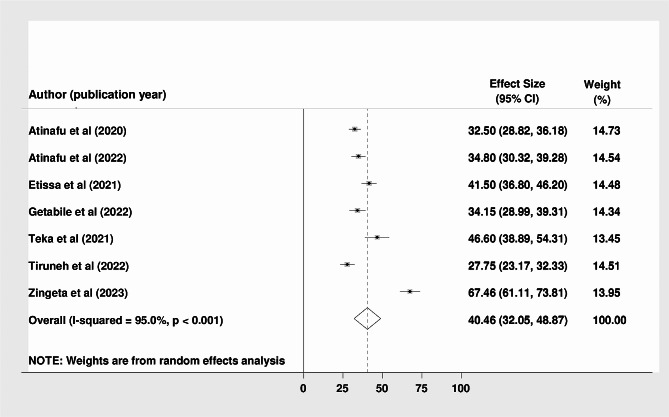
Forest plot showing the pooled mortality rate among colorectal patients in Ethiopia

The overall mortality rate among colorectal cancer patients in Ethiopia was 40.5% (95% CI: 32.05%, 48.87%; I^2^ = 95.0%; p-value < 0.001) **(**Fig. 2**).** The pooled median follow-up time across the studies was 28.74 months, and the pooled survival rates at 1, 2, 3, 4, and 5 years were estimated to be 82.3% (95% CI: 73.33, 91.31), 63.3% (95% CI: 51.24, 75.45), 48.8% (95% CI: 43.35, 54.32), 33.1% (95% CI: 30.04, 36.22), and 26.6% (95% CI: 21.26, 31.91) respectively.

### Subgroup analysis

In order to address the observed heterogeneity in the study (I^2^ = 95.0%), the subgroup analysis was conducted based on the study year, sample size, and year of publication. Subgroup analyses based on study year showed that the rate of death among colorectal cancer patients was higher in studies done in or after 2017 than in studies studied before 2017 (43.0% versus 38.2%). Similarly, when analyzing the results based on sample size, it was found that studies with a sample size of less than 384 had a higher mortality rate for colorectal cancer compared to studies with a sample size of 384 or more (43.9% versus 36.1%). However, significant variation was not observed in the rates of colorectal cancer deaths between studies published before or in 2021 compared to those published after 2021 (39.71% before/in 2021 versus 40.91% after 2021).

### Predictors of colorectal cancer mortality

In order to identify the main predictors of mortality in patients with colorectal cancer, this systematic review and meta-analysis considered variables that were associated with the risk of mortality in two or more primary studies using a multivariable Cox regression model. Accordingly, the patient’s age, marital status, comorbidities, stage of cancer, and baseline CEA level were the most common predictors of mortality among colorectal cancer patients in Ethiopia.

Older patients, aged 70 and above, were nearly 2 times more likely to die as compared to those under 40 years old (AHR: 1.89, 95% CI: 1.27–2.82). Married CRC patients had a 2.5 times higher risk of death compared to single patients (AHR: 2.53, 95% CI: 1.79–3.57). Patients with CEA levels ≥ 5ng/mL during diagnosis had a 2.06 times higher risk of death compared to those with < 5ng/mL CEA level (AHR: 2.06, CI: 1.35–3.13). The hazard of CRC death was 84% higher in patients with comorbidities compared to those without any comorbidity (AHR: 1.84, 95% CI: 1.45–2.35). Moreover, when compared to patients diagnosed at clinical stage I, those diagnosed at stage IV had an 8.06-fold increased risk of death (AHR: 8.06, CI: 2.89–22.49) **(**Table 2**).**

**Table 2:** Predictors of mortality among CRC patients in Ethiopia

Variable	Authors	AHR	95% CI	Pooled AHR	95% CI of pooled AHR
Age ≥ 70	Atinafu et al.	1.7	1.02–2.90	1.89	1.27–2.82
	Atinafu et al.	2.2	1.2–4.1		
Married	Atinafu et al.	2.4	1.5–3.8	2.53	1.79–3.57
	Atinafu et al.	2.7	1.6–4.5		
CEA level of ≥ 5 ng/mL	Teka et al.	2.31	1.27–4.19	2.06	1.35–3.13
	Tiruneh et al.	1.84	1.02–3.30		
Stage IV	Atinafu et al.	17.6	6.1–50.7	8.06	2.89–22.49
	Teka et al.	2.66	1.44–4.91		
	Atinafu et al.	20.04	6.1–66.7		
	Zingeta et al.	6.05	2.28–16.02		
Stage III	Atinafu et al.	8	2.8–23.3	8.62	3.88–19.15
	Atinafu et al.	9.5	2.8–31.8		
Stage II	Atinafu et al.	3.8	1.3–11.1	4.13	1.85–9.22
	Atinafu et al.	4.6	1.4–15.7		
Comorbidity	Atinafu et al.	1.8	1.3–2.5	1.84	1.45–2.35
	Atinafu et al.	1.9	1.3–2.7		

## Discussion

Ethiopia, as part of a low-income country, is experiencing a demographic and epidemiological transition. With rapid urbanization, changes in lifestyle, and improved life expectancy, non-communicable diseases, including colorectal cancer, are becoming more prevalent [47, 48]. However, limited resources, inadequate cancer screening programs, and challenges in early diagnosis and treatment could contribute to increased mortality rates [45, 49, 50]. Therefore, this study aimed to figure out how many patients are dying from colorectal cancer in Ethiopia and what predictors are contributing to it.

In this review, the pooled median follow-up time was 28.74 months and the pooled 1, 3, and 5-year survival rate of colorectal cancer was 82.3% (95% CI: 73.33, 91.31), 48.8% (95% CI: 43.35, 54.32) and 26.6% (95% CI: 21.26, 31.91) respectively. The 1-year survival rate of patients with CRC in our study is consistent with reviews done in different parts of the world. In Sub-Saharan Africa, the rate was 74% [51], while in Iran it was around 84–85% [52, 53]. In Eastern Mediterranean Region Countries it was 88.07% [54] and in China, it was 79% [55]. This finding was also consistent with a population-based study conducted in European countries like England, Denmark, Norway, Canada, Sweden, and Australia where the 1-year survival rate of CRC patients was 74.7%, 77.7%, 82.4%, 83.5%, 83.8%, and 84.9% respectively [56].

The 3-year survival rate for CRC patients in Ethiopia was found to be 48.8%. This rate is much lower compared to the survival rates observed in other countries such as Iran 64–65% [52, 53], Eastern Mediterranean Region Countries 70.67% [54], China 72–74% [55, 57], and Brunei Darussalam 62.5% [58]. For the 5-year survival rate, Ethiopia had a rate of 26.6%, which was again lower compared to colorectal cancer survival rates in sub-Saharan Africa 43.5% [59], Iran 52–54% [52, 53], Eastern Mediterranean region 57.26% [54], China 62–68% [55, 57], Jordan 58.2% [60], German 63.0% [61], Brazil 63.5% [62], Spain 55.5% [63] and Australia 63.3% [11]. However, the 5-year CRC survival rate in African countries such as Uganda, Ghana, and Gambia was less than 13%, 16%, and 22%, respectively [64]. This rate implies that Ethiopia and other African countries have a lot of room for improvement in terms of colorectal cancer survival rates. The reason why CRC survival rates differ could be because of a bunch of reasons like differences in patient characteristics: tobacco use [65], alcohol consumption [66], obesity [67], sedentary lifestyle [68], and comorbidity [69, 70]; dietary factors (meat vs. cereal intake) [71]; screening methods [72] and physician’s knowledge and attitudes towards screening [73, 74]. Variations in survival rates can also be brought by the stage of CRC at diagnosis [75, 76], and treatment option differences [77]. Moreover, the better survival rate in other countries compared to Ethiopia might be explained because they had more advanced cancer care and benefited from a large number of local and western-trained doctors who can provide a range of cancer treatment modalities [78].

This systematic review and meta-analysis found that the combined mortality rate for colorectal cancer patients was 40.5% (95% CI: 32.05%, 48.87%). This means that, on average, two out of every five colorectal cancer patients die from the disease. This mortality rate surpasses the national average death rate for cervical cancer, which is 16.39% [79], and lung cancer, which is only 10% [80]. However, it is noteworthy that the mortality rate was similar to Malaysia’s and other Asian countries rates of 44.7% and 52.4% for colorectal cancer mortality respectively [17, 81]. On the other hand, China [2] and Canada [82] had the lowest mortality rates for colorectal cancer, which were 28.11%, and 6% respectively. In the United States, approximately 51.3% [83] and 51.4% [84] of colorectal deaths occurred within 5 years of diagnosis. The variation in mortality rates can be explained by differences in the availability and advancement of screening, diagnostic, and treatment facilities [85]. However, the increase in mortality rates in low and middle-income countries like Ethiopia may be due to limited access to treatment options and adjunctive therapy [86], with only a small percentage of patients (1.3–3.1%) receiving radiotherapy in these countries [87]. Moreover, delays in diagnosis, referral, and treatment, along with cultural beliefs and financial constraints, may have contributed to higher mortality rates in these regions [86, 88].

This review found that elderly patients were more susceptible to death than their younger counterparts. Patients aged 70 and older were nearly 2 times more likely to die as compared to those under 40 years old (AHR: 1.89, 95% CI: 1.27–2.82). This finding is supported by a study conducted in China revealed that patients aged 70 or older had a higher risk of colorectal cancer-specific death and a reduced chance of survival [89]. It is unsurprising that younger patients exhibited a decreased mortality risk, given that they often have fewer comorbidities, a lower chance of dying from other causes, and are more likely to receive aggressive and intense treatments [90]. Once more, the elevated mortality rates among the elderly, in contrast to individuals under the age of 50, can be attributed to a heightened occurrence of cardiac complications within the older group. Grosso et al., who studied a European sample of patients, discovered that cardiovascular complications in colon cancer patients aged 65 and above were twice as prevalent as in their younger counterparts [91] and it is worth noting that cardiovascular diseases are the main cause of death among colorectal cancer patients [83].

Despite previous findings indicating that being married is associated with improved survival rates in various types of cancer, such as breast [92], colorectal [93, 94], lung [95], ovarian [96], pancreatic [97], and prostate cancer [98], our review revealed that married patients had a 2.5 times higher risk of death compared to single patients (AHR: 2.53, 95% CI: 1.79–3.57). Despite the fact that being married improved social support and had positive effects on psychological well-being, screening, diagnosis, and treatment adherence [99], it should be acknowledged that cancer outcomes are affected by a mix of biological, environmental, and social factors, regardless of marital status [100–102]. Furthermore, the association between married individuals and a higher likelihood of dying from colorectal cancer can be explained by the fact that marriage often involves shared lifestyle habits, such as dietary choices, physical activity levels, and tobacco use and alcohol consumption. If one partner engages in unhealthy lifestyle, it can potentially influence the other partner to adopt similar lifestyles, which could potentially increase the risk of dying from colorectal cancer. Another possible explanation could be that many individuals marry later in life, and our study identified older age as a significant predictor for higher mortality rates. Also, as age increases the likelihood of developing comorbidities increases, which our research highlights as another contributing factor. Thus, the combination of older age and greater comorbidity rates among married individuals may explain their increased susceptibility to colorectal cancer mortality. However, we advise to conduct further research to fully comprehend the exact link between marital status and colorectal cancer mortality.

Patients with CEA levels ≥ 5ng/mL during diagnosis were 2.1 times more likely to die (AHR: 2.06, CI: 1.35–3.13) compared to those with < 5ng/mL CEA level. This finding aligns with the results of the COLOFOL clinical trial conducted in 24 hospitals across Denmark, Sweden, and Uruguay [103]. Similar results were also observed by Becerra et al., who found a 62% increased hazard of death in patients with elevated CEA levels compared to those with normal levels [104]. This is likely due to the fact that a higher preoperative CEA level indicates poor treatment response, lower survival rates, advanced cancer stage, and nodal metastasis [105–107]. All these can contribute to an increased risk of colorectal cancer-related death.

Patients with existing comorbidities had an 84% higher hazard of death (AHR: 1.84, 95% CI: 1.45–2.35) compared to those without any comorbidity. This finding was consistent across various studies conducted in countries such as Germany, Spain, Australia, and the Netherlands [69, 108–110], which revealed that comorbidities like hypertension, diabetes, myocardial infarction, chronic obstructive pulmonary disease, and asthma were strong predictors of mortality in colorectal cancer patients. The reason behind this prediction could be that comorbidities affect the physical characteristics (morphology), tissue structure (histology), differentiation, and proliferation rate of colorectal cancer [111, 112]. Furthermore, they weaken the immune system and complicate the management and treatment of the disease, ultimately leading to a higher risk of death [113].

Moreover, when compared to patients diagnosed at clinical stage I, those diagnosed at stage II, III, and IV had a 4.1-fold (AHR: 4.13, 95% CI: 1.85–9.22), 8.6-fold (AHR: 8.62, 95% CI: 3.88–19.15), and 8.1-fold (AHR: 8.06, CI: 2.89–22.49) increased risk of death, respectively. This correlation between advanced stage at diagnosis and increased mortality rate and poor survival has been observed in studies conducted in Iran, England, and Malaysia [114–116]. This can be attributed to the fact that stage I tumors are typically small and localized in the inner layers of the colon or rectum. However, as the disease advances, tumors grow larger, invade deeper into the bowel walls, and may even spread to nearby structures [117]. This increased invasion leads to more complications and challenges in treating the cancer, eventually resulting in death.

Our meta-analysis has both strengths and limitations. One strength is that being the first study of its kind, it can provide valuable insights for program planners, policymakers, and healthcare providers involved in CRC prevention, diagnosis, and treatment. It also gives clues on the quality of healthcare provided by hospitals to CRC patients. However, several limitations to our study should be considered when interpreting the findings. Firstly, some predictor variables were only pooled from two studies, which may impact the strength of the meta-analysis. Secondly, due to the limited number of cancer centers in Ethiopia, many regions have not published studies on CRC mortality rates. As a result, certain subgroup analyses, such as analyzing mortality rates based on specific study regions, were not possible. Furthermore, there was significant heterogeneity across the included studies, possibly due to variations in follow-up periods, which ranged from 17 to 39 months. Therefore, the overall pooled mortality rate should be interpreted cautiously.

## Conclusion

In Ethiopia, the mortality rate among individuals diagnosed with CRC is high, with two out of five patients dying from this disease. This has been noticed in studies published since 2017. Age, marital status, CEA level, comorbidities, and cancer stage were identified as predictors of mortality in CRC patients. Ethiopia needs to undertake various measures to lower death rates among colorectal cancer patients. This includes, early detection and screening should be prioritized, particularly for older patients, those who are married, have comorbidities, elevated CEA levels, and advanced cancer stages. Additionally, future studies need to investigate the exact association between being married and colorectal cancer mortality, along with exploring the impact of different treatment modalities on CRC mortality rates and overall patient survival.

### Implications

The findings of this systematic review and meta-analysis have several implications. Firstly, it will contribute to the existing body of knowledge by providing valuable insights into mortality rates and predictors among Ethiopian colorectal cancer patients. This will benefit healthcare professionals, researchers, and policymakers by enhancing their understanding of factors that affect patient outcomes, guiding clinical decisions, and resource allocation. Also, the findings highlight areas that require further research and contribute to the development of evidence-based strategies to improve patient care and reduce mortality rates among colorectal cancer patients in Ethiopia.

### Electronic supplementary material

Below is the link to the electronic supplementary material.

Supplementary Material 1

Supplementary Material 2

Supplementary Material 3

## Data Availability

All data relating to the present study are provided within the manuscript or supplementary information files.

## Abbreviations

AHR: Adjusted Hazard Ratio
CRC: Colorectal Cancer
CEA: Carcinoembryonic Antigen
PRISMA: Preferred Reporting Items for Systematic Reviews and Meta-Analyses
