# Supplementary material for: Mortality rate and predictors of colorectal cancer patients in Ethiopia: a systematic review and meta-analysis
Source: BMC Cancer. 2024 Jul 10;24:821. doi: 10.1186/s12885-024-12597-9 (PMC11234545; doi:10.1186/s12885-024-12597-9)
Supplement: Supplementary file 2 — Supplementary Material 2 [file 12885_2024_12597_MOESM2_ESM.docx]

**Supplementary table 2.** Newcastle-Ottawa Quality Assessment Scale for cohort studies used in this systematic review and meta-analysis

| **Authors , year** | **Selection** | | | | **Comparability**  Comparability of cohorts on the basis of the design or analysis (**) | **Outcome** | | | **Total score** | **Quality *** |
| --- | --- | --- | --- | --- | --- | --- | --- | --- | --- | --- |
|  | Representativeness of the exposed cohort (*) | Selection of the non-exposed cohort (*) | Ascertainment of exposure (*) | Demonstration that outcome of interest was not present at start of study (*) |  | Assessment of the outcome (*) | Was follow-up long enough for outcomes to occur (*) | Adequacy of follow-up of cohorts (*) |  |  |
| Atinafu et al, 2020 | * | * | * | * | * | * | * | * | 8 | Good |
| Etissa et al, 2021 | * | * | * | * | * | * | * | * | 8 | Good |
| Teka et al, 2021 | * | * | * | * | * | * | * | * | 8 | Good |
| Atinafu et al, 2022 | * | * | * | * | * | * | * | * | 8 | Good |
| Getabile et al, 2022 | * | * | - | * | * | - | * | * | 6 | Good |
| Tiruneh et al, 2022 | * | * | * | * | * | * | * | * | 8 | Good |
| Zingeta et al,2023 | * | * | - | * | * | - | * | * | 6 | Good |
