# Supplementary figures and images for: Mortality rate and predictors of colorectal cancer patients in Ethiopia: a systematic review and meta-analysis
Source: BMC Cancer. 2024 Jul 10;24:821. doi: 10.1186/s12885-024-12597-9 (PMC11234545; doi:10.1186/s12885-024-12597-9)

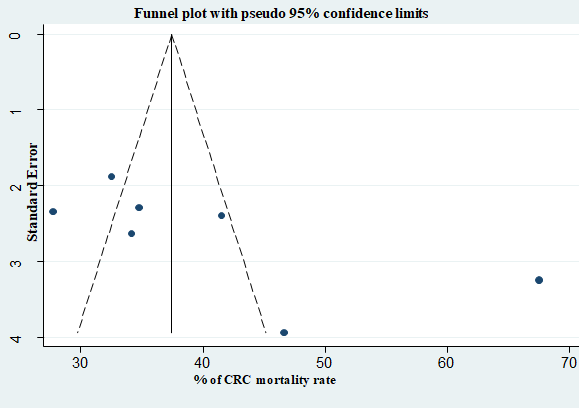


**Supplementary Figure 1:** Funnel plot for publication bias assessment

Supplement: Supplementary file 3 — Supplementary Material 3 [file 12885_2024_12597_MOESM3_ESM.docx]
